# ATP synthase and Alzheimer’s disease: putting a spin on the mitochondrial hypothesis

**DOI:** 10.18632/aging.103867

**Published:** 2020-08-27

**Authors:** Brad Ebanks, Thomas L Ingram, Lisa Chakrabarti

**Affiliations:** 1 School of Veterinary Medicine and Science, University of Nottingham, Sutton Bonington LE12 5RD, UK; 2 MRC Versus Arthritis Centre for Musculoskeletal Ageing Research, Chesterfield, UK

**Keywords:** Alzheimer's disease, mitochondria, ATP synthase

## Abstract

It is estimated that over 44 million people across the globe have dementia, and half of these cases are believed to be Alzheimer’s disease (AD). As the proportion of the global population which is over the age 60 increases so will the number of individuals living with AD. This will result in ever-increasing demands on healthcare systems and the economy. AD can be either sporadic or familial, but both present with similar pathobiology and symptoms. Three prominent theories about the cause of AD are the amyloid, tau and mitochondrial hypotheses. The mitochondrial hypothesis focuses on mitochondrial dysfunction in AD, however little attention has been given to the potential dysfunction of the mitochondrial ATP synthase in AD. ATP synthase is a proton pump which harnesses the chemical potential energy of the proton gradient across the inner mitochondrial membrane (IMM), generated by the electron transport chain (ETC), in order to produce the cellular energy currency ATP. This review presents the evidence accumulated so far that demonstrates dysfunction of ATP synthase in AD, before highlighting two potential pharmacological interventions which may modulate ATP synthase.

## INTRODUCTION

AD is a progressive, irreversible neurodegenerative disease that accounts for more than half of the 44 million cases of dementia globally [1]. AD can be either sporadic or familial (inherited). The greatest risk factor for the onset of AD is ageing, and the World Health Organization predicts that by 2050 the number of people over the age of 60 will have increased to 2 billion [2]. With that, the number of people living with AD will increase as well as the economic costs of supporting and treating AD patients.

Symptomatically, AD is initially recognised by mild cognitive impairment (MCI) and problems with short- and long-term memory. As the disease progresses neuropsychiatric symptoms can develop including affective, psychomotor, psychotic and manic syndromes [3]. There are two distinct biomolecular markers within the brain that have long been known to characterise AD, amyloid plaques composed of the amyloid-β (Aβ) peptide and neurofibrillary tangles (NFTs) composed of hyperphosphorylated tau proteins [4]. However, due to their location AD can only be diagnosed using these markers post-mortem.

Mitochondria are also widely observed as dysfunctional in AD, which has resulted in the development of the mitochondrial cascade hypothesis [5, 6]. The dysfunction of mitochondria, and in particular the ETC, has been coupled with the oxidative stress observed in AD [7, 8]. It has been widely debated as to whether amyloid plaques, NFTs or dysfunctional mitochondria play the primary role in the aetiology of AD. We now understand that interactions actually take place between these different biomolecular markers contributing to disease progression [9, 10].

When considering the role of mitochondria in AD, ATP synthase has not been widely discussed. ATPases are present across eukaryotes, prokaryotes and archaea. They can be placed into one of three different classes: F-type, V-type or A-type, similar in structure but differing in function [11, 12]. Mitochondrial ATP synthase is an F-type ATPase and is the final ETC complex of the IMM. It is responsible for the pumping of protons from the inter-membrane space into the matrix while harnessing the chemical energy from this process. The chemical energy is converted into mechanical energy that allows the complex to behave as a molecular motor. Rotation of the motor triggers conformational changes in the catalytic domain of the enzyme that enables the production of ATP, the cellular energy currency of which an estimated 50kg a day is required by the body, from ADP and P_i_[13]. In medical research, ATP synthase has been more widely studied in classic mitochondria disorders such as Leigh Syndrome [14, 15].

This review is a synthesis of the data which implicate ATP synthase in the pathology of AD. It then considers ways in which ATP synthase can be therapeutically targeted in order to try and prevent disease onset or to alleviate symptoms.

### ATP synthase

### What is ATP synthase

F-type ATP synthase is the fifth and final ETC complex of the IMM. It has a large structure with a molecular weight of around 600 kDa and is composed of up to 20 different subunits in mammals [16]. ATP synthase is responsible for the production of the cellular energy carrier ATP from ADP and P_i_. This process is driven by the chemiosmotic potential across the IMM first described by Peter Mitchell in the 1960s [17–19]. While F-type ATP synthase is predominantly housed within mitochondria, data have shown that F-type ATP synthase is present at plasma membranes of different cell types both physiologically and pathophysiologically [20, 21].

**Figure 1:**
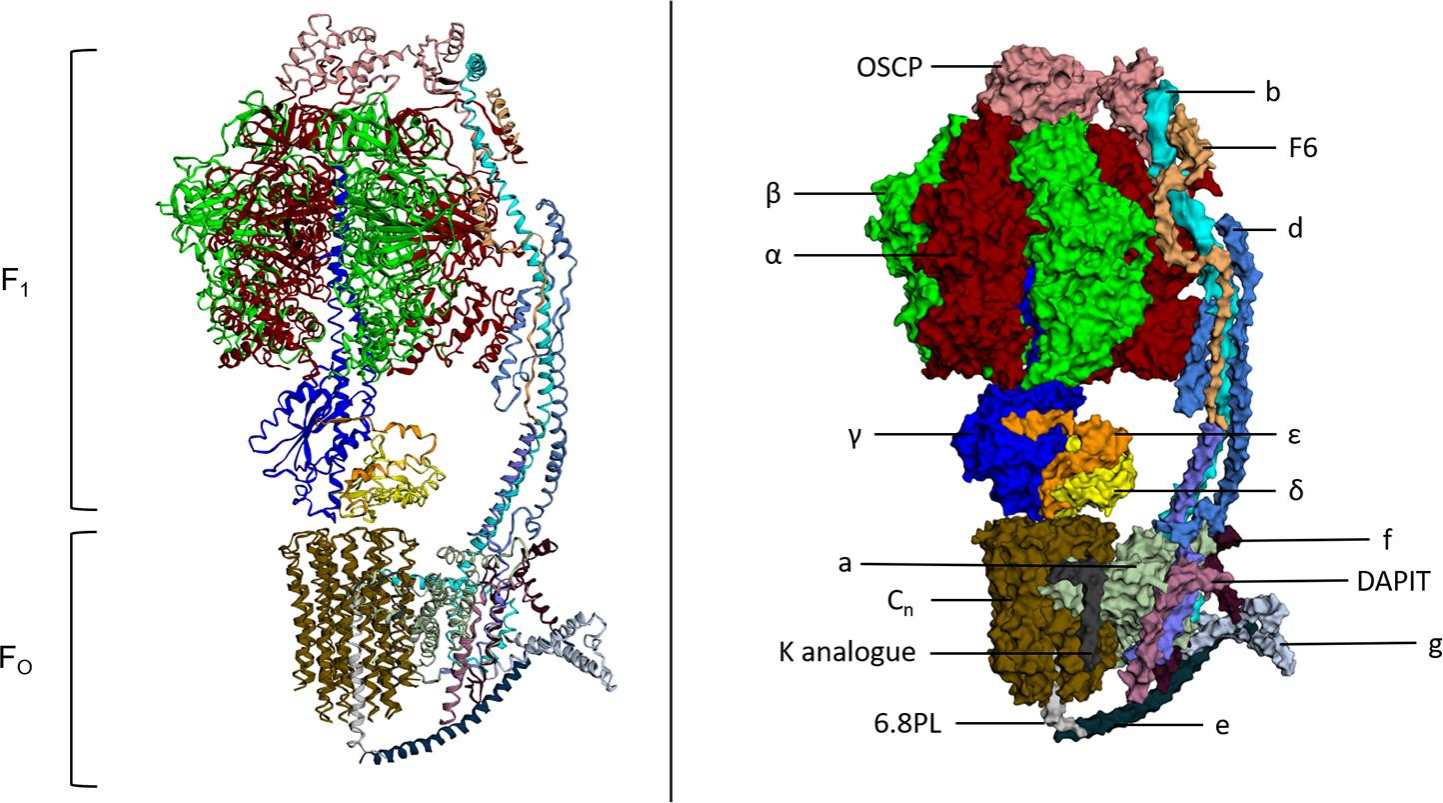
**Atomic structure and labelled space fill model of ATP Synthase (S. scrofa).** F_O_ and F_1_ components of the complex both labelled. Individual subunits labelled on the space fill model. This figure was created using image 6J5J from PDB (http://doi.org/10.2210/pdb6J5J/pdb https://www.rcsb.org/structure/6J5J) and processed using http://www.sbg.bio.ic.ac.uk/ezmol/.

After ATP synthase’s function was described *in vitro*, landmark measurements including the kinetic parameters of its three-site cooperative-binding catalytic mechanism and the discovery that protein conformational changes would facilitate the release of tightly bound ATP were reported [22, 23]. The atomic structure of the complex was resolved to 2.8 Å in 1994, revealing a structure which supported the mechanism of rotary catalysis [24]. Since 1994, multiple atomic structures of both eukaryotic and prokaryotic ATP synthase structures have been published, with a recent cryo-EM structure of ATP synthase from *S. scrofa* shown in Figure 1 [25–28]. ATP synthase consists of two distinct components; a membrane bound F_O_ component and a matrix exposed F_1_ component. They function cooperatively through a central rotor stalk and a peripheral stator stalk.

### F_O_

The F_O_ component of ATP synthase is an insoluble structure that is primarily composed of a ring of varying numbers of c-subunits called the c-ring and has a size that appears to be species dependent [29]. Other F_O_ proteins include subunit a and subunit b as well as others with less well understood roles including subunits d, e, f, g, F6 and 8 (A6L). DAPIT and 6.8PL are present in vertebrates and assist in the assembly of the F_O_ component of ATP synthase [30, 31]. An additional subunit called the oligomycin sensitivity conferring protein (OSCP) is located at the top of the F_1_ component of ATP synthase. It couples the F_O_ component with the F_1_ component through its interaction with the peripheral stalk of F_O_ and central stalk of F_1_ [32]. Protons from the intermembrane space of the mitochondria travel through an aqueous half-channel in subunit a to the c-ring of the F_O_ complex where they bind to conserved acidic c-ring residues, aspartate or glutamate, in the second transmembrane helix of subunit-c [13, 33–35]. These charged proton binding sites are then suggested to be concealed by rotation of α-helices in c subunits which leads to c-ring to rotation along with the central rotary stalk γ-subunit [36, 37]. The rotating F_O_ component transports protons into the matrix through a second aqueous half channel on the matrix side of the membrane and the asymmetric rotor stalk causes conformational changes in F_1_ which drive the catalytic activity of the β subunits [13].

### The role of OSCP

The oligomycin sensitivity conferring protein (OSCP) is part of the peripheral stalk of the F_O_ component of ATP synthase and physically couples the two enzyme components together through its interaction with the central stalk of the F_1_ component. It is encoded by the *ATP5O* gene on the long arm of the nuclear chromosome 21. Structurally, OSCP has an N-terminal domain which contains six α-helices and a C-terminal domain consisting of a β-hairpin and two α-helices [32, 38]. While oligomycin does not bind to OSCP, OSCP confers the enzyme’s sensitivity to the antibiotic as it is OSCP that couples the F_1_ component to the F_O_ component that is bound and inhibited by oligomycin [39].

### F_1_

**Figure 2:**
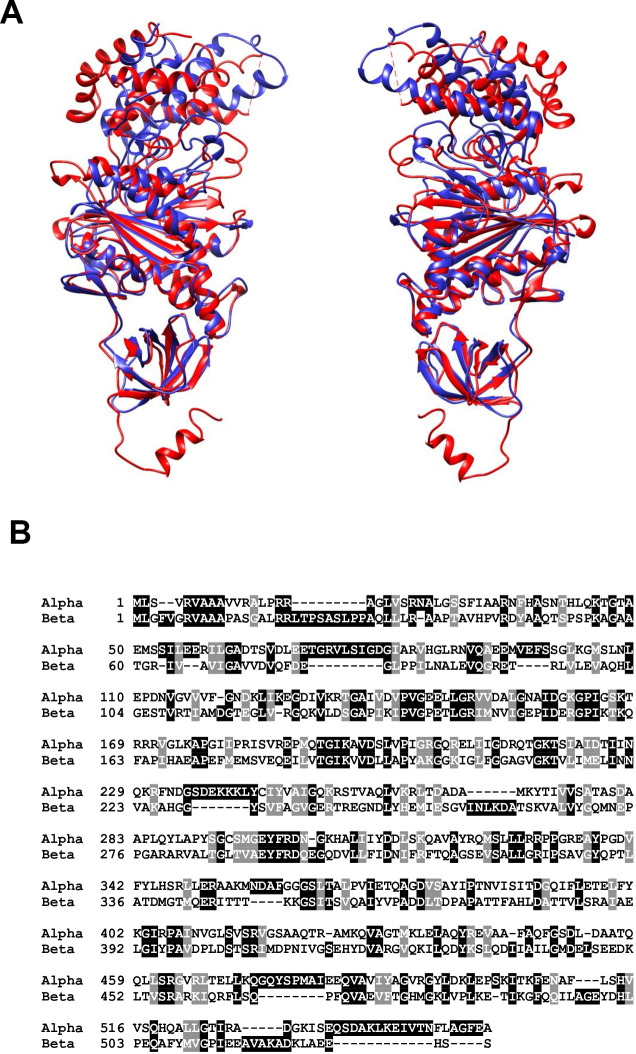
(**A**) Structural alignment of the alpha (red) and beta (blue) subunits of mitochondrial ATP synthase in S. scrofa. Both subunits are reproduced from image 6J5J in PDB (https://www.rcsb.org/structure/3ZIA http://doi.org/10.2210/pdb3ZIA/pdb) and processed using http://www.cgl.ucsf.edu/chimera/. (**B**) BLAST alignment of the primary amino acid sequences of H. sapiens alpha (UniProt P25705) and beta (UniProt P06576) subunits performed using https://blast.ncbi.nlm.nih.gov/Blast.cgi?PAGE=Proteins.

The F_1_ component is solvent exposed and far more about its activity and structure is understood than its F_O_ counterpart. Its subunit composition is α_3_β_3_γδε, with its structure being a six part barrel of alternating α and β subunits, a central asymmetric γ subunit (the aforementioned rotor stalk) protruding through the centre of the barrel while the δ and ε subunits are found at the matrix exposed surface of the F_O_ c-ring [24]. The site of catalysis is located at the interface of the α and β subunits, both of which have nucleotide binding sites and multiple studies of atomic structures have shown nucleotides bound at this interface [40–42]. Interestingly, both α and β subunits possess the same folds despite only sharing around 20% sequence homology [16]. The structural similarities are presented in Figure 2 using the atomic structures of ATP synthase in *S. scrofa* [27, 43]. Despite the similarity, only the β-subunit possesses catalytic activity due to its ability to form an open conformation as well as possessing a catalytic base for the reverse ATP hydrolysis reaction [44, 45].

### The role of the α-subunit

The α-subunit of the F-type ATP synthase is located in the F_1_ solvent exposed component of ATP synthase, facing the mitochondrial matrix [24]. It functions as part of a six-part barrel structure (α_3_β_3_), and the catalytic nucleotide binding site is located at its interface with the β-subunit [40]. However, the α-subunit displays regulatory activity when compared with the β-subunit which exhibits the catalytic activity of the enzyme [44, 45]. Like its β-subunit counterpart, the α-subunit can be divided into three different domains: a small N-terminal domain, a nucleotide binding domain and a helical C-terminal domain.

### The role of the β-subunit

The β-subunit is also located in the solvent exposed F_1_ component of ATP synthase and it has a largely similar structure to the α-subunit. Its interface with the α-subunit forms the nucleotide binding site and it is the β-subunit that possesses the catalytic activity required for both the synthesis and hydrolysis of ATP. The β-subunit is able to undergo conformational changes to form three distinct conformations in response to the rotation of the γ-subunit in 120° increments [46]. This, coupled with critical arginine, lysine and glutamate residues is what enables β-subunit to catalyse the synthesis and hydrolysis of ATP [47]. For well-illustrated figures of this mechanism, see Feniouk et al., 2008 and Okuno et al., 2011 [48, 49].

### ATP synthase in Alzheimer’s Disease (AD)

Mitochondria are known to be dysfunctional in AD patients and this has resulted in the development of the mitochondrial cascade hypothesis [5]. This hypothesis has been developed and revisited several times across the last two decades, serving as a viable alternative to the predominant amyloid hypothesis [6, 50–52]. Much of the focus of the research has been based on the oxidation levels found in the brains of AD patients, and how this observation can be synthesised with the mitochondrial theory of ageing [4, 53]. Despite its physiological relevance to both mitochondrial activity and structure, little attention has been paid to ATP synthase in the formation and development of this theory [54].

The first study implicating ATP synthase in AD aetiology found, through BN-PAGE analysis, decreased expression of the whole complex in the hippocampal tissue of AD patients [55]. Since then, multiple studies have pointed to a decrease in the expression of ATP synthase subunits and they are addressed in this review. There was decreased expression in several of the nuclear encoded ATP synthase genes in the posterior cingulate cortex (11), hippocampal field CA1 (10), middle temporal gyrus (9) and entorhinal cortex (5) [56]. Adult neurogenesis defects are common in AD and it has been suggested that this arises from impaired function of hippocampal neuronal stem cells (NSCs). A study using iPSC-derived NSCs, with familial AD (FAD) associated PS1 mutation M146L, observed a decreased expression of the ATP synthase complex while PS1 expression was kept at physiological levels [57]. In a study with implications for sporadic AD, N2a neuroblastoma cells expressing the ApoE4 allele of the ApoE gene, the major genetic risk factor for sporadic AD, showed a reduction in the levels of all ATP synthase subunits they detected in comparison to ApoE3 controls [58].

Considering ATP synthase activity instead of protein expression, an early study investigating AD and ATP synthase found no significant decrease in the enzyme’s catalytic activity when studying the isolated mitochondria from AD patient hippocampal tissue, motor cortex and platelets [59]. However, since this study was published there have been data published that are contradictory to this observation and these are discussed in the following sections of this review.

### The α-subunit in AD

### ATP synthase subunit α, Amyloid β and NFTs

Transgenic Swedish APP mice (Tg2576) had increased levels of amyloid plaque formation in the brain as they aged, compared with controls. Proteomic analysis of the brains from the Tg2576 mice found that the increase in amyloid plaque deposition with age correlated with an increase in the expression of the α-subunit [60].

An N-glycosylated form of the α-subunit has been shown to act as a binding partner of the extracellular domain of APP and Aβ, with Aβ being the primary component of the AD hallmark amyloid plaques. The α-subunit reaches the membrane via the secretory pathway and it is during this process that it becomes N-glycosylated. Schmidt et al. also demonstrated the localisation of the whole ATP synthase complex at the neuronal membrane and that its extracellular ATPase activity is inhibited by both APP and Aβ. This is especially noteworthy as APP and Aβ share sequence homology with the native ATPase inhibitory factor IF1 [61]. The inhibition was shown to downregulate long-term potentiation (LTP) at the synapses, that Aβ oligomers have since been shown to inhibit alongside the upregulation of long-term depression (LTD) via the NMDA receptors [62].

A study that builds upon the work of Schmidt et al. found that in the cortex and hippocampus of Tg APP PS1 mice, the α-subunit co-localises with insoluble plaques of Aβ – not just the soluble monomeric form of the peptide [63]. Moreover, the authors showed that this interaction occurs at the plasma membrane of neuronal cells, causes inhibition of enzymatic activity and a decrease in the levels of extracellular ATP. These decreases of extracellular ATP may be critical in the cognitive defects which arise in AD due to disruptions in synaptic plasticity, given the important role extracellular ATP plays in LTP [64, 65].

The α-subunit has been observed as part of the NFTs in human AD patient brain samples, one of the characteristic observations in AD patient brains. Monoclonal antibodies that target the insoluble brain lesions in AD found that the α-subunit acted as an antigen to one of the antibodies (AD46). Immunohistochemistry and electron microscopy confirmed the co-localisation of the α-subunit with the NFTs in the cytosol of a degenerating AD neuron [66].

### ATP synthase subunit α and oxidative stress

Oxidative stress is a frequently observed phenomenon of AD. *C. elegans* that over-expressed green fluorescent protein (GFP) as a means of studying the oxidative stress caused by protein aggregation presented carbonylation of the α-subunit [67]. In the hippocampus of AD patients the α-subunit was also shown to be excessively nitrated in comparison to age-matched control brains, as well as having significantly increased protein levels [68].

Another marker of oxidative stress is the level of lipid peroxidation, that arises from the reaction of oxygen radicals with lipids to produce reactive aldehydes. One such example of this is 4-hydroxy-2-nonenal (4-HNE), that covalently attaches to proteins in a Michael addition reaction [69]. The α-subunit of ATP synthase was shown to be HNE modified in the hippocampal tissue of individuals with mild cognitive impairment (MCI), which is symptomatic of early stage AD [70, 71]. The same study also showed that, in the same tissue from MCI patients, ATP synthase had a 35% decrease in activity compared to age-matched controls when measured as a function of ADP production.

A study investigating oxidative stress in the early stages of AD (Braak stages I and II, prior to the onset of MCI) found that the α-subunit of mitochondrial ATP synthase is HNE modified in the entorhinal cortex and that ATP synthase has a decrease in activity of around 30% [72, 73]. The authors chose to use the entorhinal cortex tissue for this study as it is the location of NFTs used to track AD progression during Braak stages I and II. When these data are taken with those from Reed et al. showing 4-HNE modification of the α-subunit and decreased ATP synthase activity, there appears to be correlation between disease progression as measured by the presence of NFTs and the lipoxidation of the α-subunit resulting in reduced ATP synthase activity. Further, the presence of oxidative stress and diminished ATP synthase activity from the earliest stages of AD onset may prove critical to the pathology of the disease. If this oxidative stress precedes the presence of Aβ in the affected tissue, it raises additional questions about the primacy of the amyloid pathology in the aetiology of AD.

Whether or not the α-subunit is oxidised may be dependent upon the tissue that it is found in and the stage of AD pathology in which it is being considered. A line of transgenic mice (J20 Tg) expressing a mutant form of APP that corresponds to the Swedish and Indiana familial forms of AD had a 12.2-fold increase in the expression of the α-subunit in a whole mouse brain homogenate compared with non-Tg mouse brain homogenate [74]. However, there was no indication of oxidation when measured as a function of 3-nitrotyrosine (3-NT) modification of the protein. The authors suggest that this significant increase in the expression of the protein could be a related to cellular stress responses by the brain to maintain energy production. Future studies should look to measure the α-subunit expression of early, middle and late Braak stages of AD in brain tissues shown to have reduced ATP synthase activity as a way to try and validate their suggestion.

### Post-translational modification of the α-subunit

Glycosylation of proteins with O-linked β-N-acetylglucosamine (O-GlcNAc) is a widely observed post-translational modification that regulates intracellular events [75]. The α-subunit can be O-GlcNAcylated on the Thr432 residue. However, this modification is reduced in the brains of AD patients, Tg AD mice and in Aβ treated mammalian cell cultures – which resulted in reduced ATP levels [76]. Molecular modelling and co-IP experiments with deletion mutants of the α- and β-subunits with no pocket site showed that Aβ directly blocks the O-GlcNAcylation of the Thr432 residue by mitochondrial O-GlcNAc transferase. Interestingly, the O-GlcNAcylation of Thr432 that had been inhibited by Aβ was rescued by treatment with O-GlcNAcase inhibitor. These findings are particularly noteworthy as they demonstrate a chemical mechanism for the interaction of the Aβ peptide with mitochondrial ATP synthase, and as a result could offer a potential therapeutic target for AD.

### The β-subunit in AD

### Downregulation of the β-subunit

Several studies have found changes in the expression of the β-subunit of ATP synthase in AD tissue samples and models of AD, and in particular reductions in its expression. Table 1 lists changes in protein expression of ATP synthase subunits, including the β-subunit, that are presented in this review. An early observed instance of reduced expression is the reduction of β-subunit mRNA levels by over 50% in the midtemporal cortex of AD patient brains compared with age-matched controls [77]. In another study, that linked Aβ peptides with ATP synthase in AD, rats that received a bilateral intrahippocampal injection of Aβ showed a significant decrease in the levels of β-subunit compared with controls [78]. Gene expression analysis of the entorhinal cortex of AD patient brains showed reduced expression of *ATP5C1* (γ-subunit), *ATP5D* (δ-subunit), *ATP5G1* (subunit c) and *ATP5B* (β-subunit) [79]. This strengthens the argument that ATP synthase dysfunction plays a role in the disrupted glycometabolism of AD. It must be noted that these studies do not provide a mechanism of how the expression of the β-subunit is downregulated, but early gene mapping studies of the β-subunit reported that ETS domain transcription factors and redox sensitive OXBOX and REBOX transcription factors regulate gene expression [80–82].

**Table 1:** Regulation of individual ATP synthase subunit protein expression levels across different tissue samples from different AD models, summarising data presented in this review.

**Model**	**Tissue**	**α-subunit**	**β-subunit**	**OSCP**	**Subunit d**	**δ-subunit**	**Reference**
Aβ injected rat	Hippocampus		Down				Shi, X. et al., 2011
SweAPP Tg mice	Whole brain homogenate	Up					Carrette, O. et al., 2006
4 months old 5xFAD mice	Synaptic mitochondria			Down			Beck, S. J. et al., 2016
9 months old 5xFAD mice	Synaptic mitochondria			Down			Beck, S. J. et al., 2016
Non-synaptic mitochondria				Down			Beck, S. J. et al., 2016
3x Tg AD mouse	Hippocampus				Down		Yu, H. et al., 2018
AD patient	Temporal lobe			Down			Beck, S. J. et al., 2016
	Medial frontal gyrus				Down		Adav, S. S. et al., 2019
	Temporal cortex				Down		Mukherjee, S. et al., 2017
	Frontal cortex					Up	Manczak, M. et al., 2004

### Autoimmune response to the β-subunit

Autoimmunity is now thought to play a role in the onset of AD [83, 84]. While this hypothesis has not been developed to the same extent as the amyloid, tau and mitochondrial hypotheses, the idea is grounded in the fact that anti-neuronal antibodies have been found in the sera of AD patients. Notably, it was found that the brain of AD patients contains antibodies which target the c-terminal domain of the β-subunit [85]. In neuroblastoma cell lines these antibodies caused a dose dependent decrease in the activity of the ATP synthase complex, and then most strikingly, apoptosis. The apoptotic event was preceded by IMM hyperpolarization and then depolarization.

A study that followed this showed that mice injected into their right cerebral ventricle with anti-β-subunit antibodies isolated from AD patient sera had reduced memory retention [86]. Additionally, an increased rate of apoptosis was detected in the dorsal hippocampal regions of their brains, post-mortem. Taken together, these two studies show a mechanism for the antibodies detected in the brain sera of AD patients to cause neuronal apoptosis and cognitive impairment, both of which are classical symptoms of AD.

### Excitotoxicity and cyclin-B1 accumulation

Excitotoxicity is common to neurons in AD and may be mediated by the action of glutamate on the NMDA receptors of excitatory post-synaptic neurons [87]. Interestingly, a mechanism of ATP synthase inhibition via action on the β-subunit has been elucidated in rat cortical neurons and HEK293T cells using glutamate induced excitotoxicity [88]. Cell cultures of rat cortical neurons were treated with glutamate which resulted in an accumulation of cyclin-B1, the cyclin-B1 was shown to form complexes with Cdk1 which accumulated in mitochondria resulting in superoxide production. HEK293T cells were then used in the study to demonstrate that the cyclin-B1-Cdk1 complex phosphorylates Bcl-xL causing its dissociation from the β-subunit of the ATP synthase, a reduction in the enzyme’s catalytic activity and increased oxidative stress. Bcl-xL is a transmembrane mitochondrial protein that acts as a regulator of cell death through its action on proapoptotic factors [89]. Bcl-xL has also been shown to improve the efficiency of neuronal metabolism through its interaction with ATP synthase which decreases membrane-ion leakage [90]. From these data it is clear that a disruption of the interaction between the β-subunit and Bcl-xL could contribute to AD pathology.

### OSCP in AD

### OSCP downregulation in AD

In 2016 a comprehensive study was published investigating changes in expression of OSCP in the brains of human AD patients, MCI patients and Tg AD mice brains (5xFAD mice) [91]. OSCP was shown to be significantly downregulated in the temporal lobe of AD patients compared to controls. There was also a significant decrease in OSCP expression between the synaptic mitochondria of young and old 5xFAD mice compared with controls as well as in the non-synaptic mitochondria of old 5xFAD mice. Primary cultured mice neurons with downregulated OSCP showed decreased membrane potential, reduced ATP synthesis and elevated levels of superoxide. Beck et al. also demonstrated that there is a physical interaction between Aβ and the OSCP in brain mitochondria which reduced ATP synthase activity, which is supported by evidence of Aβ localising to brain mitochondria [92, 93]. This study is notable as it provides mechanistic detail and also presents another case of Aβ peptides interacting with ATP synthase subunits in a detrimental fashion, as is the case with the α-subunit.

### Interaction with Cyclophilin D (Ppif)

Cyclophilin D (CypD) is one of the only proteins which appears to be essential to the elusive molecular make-up of the mitochondrial permeability transition pore (mPTP) [94]. CypD has also been demonstrated to interact with ATP synthase, regulating the formation of the respiratory efficiency enhancing synthasome [95]. Of note, it was also shown that synthasome assembly and mPTP formation are inversely proportional. A study of mice in 2017 found that CypD levels increased with ageing, as did the physical interaction between OSCP and CypD, despite a decrease in the expression levels of OSCP [96]. These changes resulted in decreased ATP synthase activity and an increase in mitochondrial dysfunction, including a decreased ATP:oxygen ratio. A follow up study found that the temporal lobe of AD patient brains and 5xFAD mice had increased formation of CypD-OSCP complexes, and that the presence of Aβ substantially decreased the K_D_ of this interaction [97]. The authors also showed that in 5xFAD mice CypD promotes the OSCP-Aβ interaction as well as the ubiquitin mediated degradation of OSCP. However, CypD deficient 5xFAD mice had improved cognitive function and attenuated ATP synthase deregulation compared to their 5xFAD littermates.

### Subunit d in AD

### Decreased expression and gene locus risk factor

Subunit d of mitochondrial ATP synthase is a component of the F_O_ peripheral stalk which is encoded by the *ATP5H* (*ATP5PD*) gene, located on the long arm of nuclear chromosome 17. A genome wide association study (GWAS) found that the shared locus of *ATP5H* and *KCTD2* could be a genetic risk factor for AD, where until a few years ago *APOE4* was thought to be the only instance of this [98]. A study of 3x Tg AD mice found significantly decreased expression of *ATP5H* in hippocampal tissue [99]. An LC-MS/MS-based iTRAQ quantitative proteomics study also demonstrated that multiple proteins from the mitochondrial proteome are under-expressed in the medial frontal gyrus of AD human patients including *ATP5H*, *ATP5B*, *ATP5I* and *ATP5J* compared with age-matched controls [100]. Perhaps most interestingly, another GWAS found the *ATP5H* gene to be a candidate gene of interest in late-onset AD (LOAD) and that its expression was decreased in the temporal cortex of AD patients [101]. An RNAi knockdown of *C. elegans* Tg for Aβ peptide proved to be protective against Aβ toxicity. From these data we can see that the *ATP5H* gene appears to be associated with LOAD, but any kind of molecular mechanism for this association is yet to be elucidated.

### The δ-subunit in AD

### Upregulation in AD

The δ-subunit of mitochondrial ATP synthase is part of the F_1_ component and associates with the γ-subunit of the rotary stalk, in proximity of the F_0_ c-ring. It is encoded by the *ATP5D* gene located on the short arm of nuclear chromosome 19. In 2004 a study from Manczak et al. showed increased mRNA levels for *ATP6* and *ATP8* genes in AD patient brains, while immunofluorescence analysis of the frontal cortex of AD patients found increased levels of the δ-subunit of ATP synthase [102]. While isolated, these data show yet another example of altered patterns of subunit expression across different tissues of the human AD brain.

### ATP synthase therapeutics in AD

### J147

J147 was identified in 2011 through a drug discovery scheme that sought to target age associated pathologies, as opposed to amyloid plaques, due to age being the greatest risk-factor in AD onset [103]. J147 is a neurotrophic compound that has proven safe to use in animal studies and has been shown to rescue cognitive defects in aged mouse models of AD [104]. The cognitive rescue effects seen in this study are shown to correlate with the induction of the neurotrophic factors NGF (nerve growth factor) and BDNF (brain derived neurotrophic factor). A later study from the same group demonstrated that the α-subunit is a molecular target of J147 and that J147 modulates ATP synthase activity [105]. The mild inhibition of ATP synthase by J147 may be neuroprotective. J147 also activates the canonical longevity pathway of AMPK/mTOR via CamKK2 and its administration was shown to extend the lifespan of *Drosophila*. This is noteworthy due to the fact that ageing is the biggest risk factor for the onset of AD.

Following this, a computational modelling paper of mitochondrial αβγ was published which demonstrated a mechanism of how J147 could bind to the α-subunit and modulate enzymatic activity [106]. Soliman et al. then used their per-residue energy decomposition (PRED) protocol to identify three compounds from a molecular library which could modulate ATP synthase activity in a similar manner to J147 [107]. The compounds which they identified had a higher binding propensity for the α-subunit than J147 and specifically targeted Arg1112 and Gln426 for binding.

While J147 may have potential as an effective treatment for AD, it is noteworthy that the mechanism of action is through an inhibition of ATP synthase activity given that ATP synthase activity inhibition has been observed as part of the pathophysiology of AD. It may be that there are subtle but significant differences in the decreased levels of enzymatic activity between those induced by J147 and those observed in studies of AD. We suggest that the stage of disease progression should also be considered with J147 administration, as it may be the case that the positive outcomes of J147 treatment may not be observed once pathological ATP synthase activity inhibition crosses a certain threshold.

Taken together, these data present J147 as a potentially suitable AD drug which alleviates cognitive symptoms after they have presented with a known mechanism of action. Currently J147 is undergoing clinical trials to assess its safety and efficacy as a treatment for AD.

### Salvianolic acid B (SalB)

Salvianolic acid B (SalB) is a polyphenolic compound which possesses therapeutic potential as a treatment of AD. SalB has been suggested to act on multiple different pathologies present in various neurodegenerative diseases, and in particular mitochondrial dysfunction [108]. In both cellular and mouse models of AD it has been reported that SalB can inhibit Aβ generation and may help to prevent neuroinflammation [109–111]. Alongside mitochondrial dysfunction, these two phenomena are pathologies classically associated with AD.

With regards to ATP synthase and SalB, a study in 2018 showed that in mouse neuronal cell cultures treated with Aβ SalB was able suppress superoxide production, preserve mitochondrial dynamics and mitigate the decrease in ATP synthase activity [112]. While no mechanism is offered by the authors of the paper, this is a line of investigation that we believe should be further pursued.

## CONCLUSION

AD is widely studied due to the hugely debilitating effects it exhibits on the individual, as well as its prevalence in countries with ageing populations. The dysfunction of mitochondria is heavily implicated in the aetiology of the sporadic and familial forms of AD. There is some debate about whether mitochondrial dysfunction is the primary lesion in the disease onset. Likely mitochondrial dysfunction is a convergence point for several concurrent lesions resulting in disease pathology and progression. However, little attention has so far been paid to the role that ATP synthase may play in AD. The data presented in this review suggest that this is an oversight and that the dysfunction of ATP synthase and its constituent components not only leads to disease onset, but that the enzyme complex can be targeted pharmacologically to treat the disease. In J147 there is a candidate drug currently undergoing clinical trials, and we follow these developments with cautious optimism. While only one study so far has investigated the efficacy of SalB as a potential therapeutic agent for AD, the data produced is encouraging and we hope to see this investigated further. Due to both the structural and functional complexity of ATP synthase, we see that its contribution to both disease pathology and its potential therapeutic targeting should be considered with enthusiasm and studied with intellectual nuance.
